# Reinvestment – the Cause of the Yips?

**DOI:** 10.1371/journal.pone.0082470

**Published:** 2013-12-05

**Authors:** Martin Karl Klämpfl, Babett Helen Lobinger, Markus Raab

**Affiliations:** 1 Department of Performance Psychology, Institute of Psychology, German Sport University Cologne, Cologne, North Rhine-Westphalia, Germany; 2 Department of Applied Sciences, London South Bank University, London, United Kingdom; Tokyo Metropolitan Institute of Medical Science, Japan

## Abstract

The yips is a multi-etiological phenomenon consisting of involuntary movements during the execution of a skill (e.g., a golf putt). Reinvestment, the conscious control of a movement that detrimentally affects automated movements, is thought to be a potential mechanism leading to the yips. Preventing yips-affected golfers from consciously controlling their movement, therefore, should be beneficial. The aim of the study was to be the first to empirically test in a laboratory whether reinvestment causes the yips and to explore if the tendency to reinvest can explain yips behavior. Nineteen yips-affected golfers participated in a lab experiment. They putted with the dominant arm in a skill-focus and an extraneous condition, in which they had to perform different dual tasks designed either to direct their focus on their own skill or to distract them from it. The tendency to reinvest was estimated via the Movement-Specific Reinvestment Scale. Yips behavior was assessed by putting performance and movement variability. Although the dual-task performance showed that the attentional manipulation worked, the tendency to reinvest did not predict the behavior of the yips-affected golfers in either putting condition. The yips-affected golfers also showed no difference in yips behavior between the skill-focus and the extraneous condition. In other words, the attentional manipulation did not change yips behavior. The data do not support the assumption that there is a link between the yips and reinvestment, likely because of the multi-etiological nature of the yips. Other psychological or neurological mechanisms such as conditioned reactions may better explain the yips and should be investigated.

## Introduction

Putting is one of the most important strokes in golf and has received extensive attention in research (e.g., 1). Like all precision sports (e.g., bowling, archery, and darts), golf requires accurate movements to direct an object toward a goal location [2]. Some famous golf players, such as Bernhard Langer, struggled in their careers because they suffered from the putting yips, which does not allow the execution of accurate movements. The yips is defined as the occurrence of involuntary movements during the execution of a fine motor skill [3] and it is a common phenomenon, with a reported prevalence ranging from 28 to 48% [3,4]. These involuntary movements, usually twisting and jerks of the wrist and lower arm shortly before hitting the ball, disturb the execution of a smooth, controlled putt, resulting in poor performance [3–5]. The putting yips is proposed to be measured by the estimation of the performance and the movement variability over repeated trials of putts with the dominant arm from a short distance [6]. Yips-affected golfers show a higher movement variability and a lower performance compared to unaffected golfers [6,7]. In this context, movement variability cannot be seen as functional like in research about dynamical systems theory [8], in which a high variability represents the ability of highly skilled athletes to adapt complex motor patterns to various performance situations. In yips research, movement variability expresses the uncontrollability of the movement caused by the yips. The etiology of the yips remains unclear. In the following, we present an overview of contemporary beliefs about the etiology of the yips and then introduce reinvestment as a possible yips mechanism in more detail.

### Yips Etiologies

Different yips etiologies have been reported in the literature. The predominant model is Smith et al.’s [9] continuum model, which places yips on a continuum anchored by a neurological and a psychological origin. It assumes that both neurological and psychological mechanisms have explanatory power, but their proportion of contribution to the occurrence of the yips can vary within the affected athlete, and this determines the type of yips. Type I describes athletes who exhibit mainly neurological symptoms associated with task-specific focal dystonia. This neurological movement disorder is defined as involuntary muscular contractions that lead to repetitive movements or abnormal postures of one body part, which occur exclusively during the execution of one specific task [10]. Type II characterizes the yips driven by mainly psychological mechanisms related to choking under pressure (choking). Choking is defined as the “process, whereby the individual perceives their resources are insufficient to meet the demands of the situation, and concludes with a significant drop in performance—a choke” [11]. An alternative explanation that has received less attention was provided by Marquardt [7]. He claimed that the yips is a contextual movement disorder learned by fatal movement or technique strategies. Here, the yips can be seen as a conditioned reaction that occurs only under specific circumstances. For instance, when ball is removed, no yips appeared during the putting swing. In addition, previously learned movements from other sports, such as the tennis stroke, might have an influence on the development of the yips in golf and further explain why novices can be affected by the yips [7].

Empirical evidence to support the validity of these etiologies is generally missing. The present study, however, focused on yips being caused by psychological mechanisms because Klämpfl et al. [6] did not find a strong connection between the yips and the task-specific focal dystonia in their sample.

### The Yips and Reinvestment

There are indices of the relevance of reinvestment as a mechanism for the occurrence of the yips in previous studies. First, in an interview-based study, some yips-affected golfers reported focusing on the skill when executing the putt [12]. Second, Bawden and Maynard [13] related the yips to a severe form of choking based on statements of yips-affected cricketers in an interview-based study. Third, Klämpfl et al. [6] associated the yips with a chronic and severe form of choking, referring to a stably occurring phenomenon that underlies choking mechanisms. In contrast to the original definition of choking that sees it as an acute response to anxiety induced by pressure, no pressure is needed to provoke the performance decrements in the yips. The yips also occurs in training sessions, where the pressure is generally low or nonexistent [3]. Performance anxiety is reported to play an important role in the occurrence of the yips [3,4]. Therefore, the yips could be rather seen as situational choking, in which the situation itself, putting the ball, provokes performance anxiety. One relevant mechanism similarly reported to be responsible for the appearance of a choke and the yips is described in reinvestment theory [14]. While there is considerable evidence for the relevance of reinvestment in the occurrence of a choke [14], empirical support for reinvestment causing the yips is missing.

Reinvestment is defined as the attempt to consciously control one’s own movement during skill execution by the application of explicit and rule-based knowledge [15]. As also Masters & Maxwell [14] described, the statement of a yips-affected cricketer in an interview-based study illustrates the relevance of reinvestment for the yips’ occurrence [13]: ‘As I got to the top of my run, I just thought “how do I let get of the ball”… and suddenly the ball was stuck in my hand…’. A prerequisite of reinvestment is the acquisition of explicit knowledge. Furthermore, the skill has to be in the learning stage, where it has already been transferred to the implicit system and runs automatically. Novices, who still rely on explicit processes during the execution of a skill, do not experience performance decrements when provoked to reinvest [16–18]. In contrast, the performance of skilled athletes suffers from reinvestment, which breaks down the automated skill into smaller parts as in the early stages of learning, increasing the chance to produce errors [19]. Different contingencies have been reported to cause the athlete to reinvest [14]. For instance, reinvestment in athletes can occur in pressure situations, as a consequence of injury, accident, or movement disorder, in situations where they have to adapt to new circumstances (e.g., new sports equipment), and after unexpected performances or events. Significant life events seem to be connected with the onset of the yips, such as humiliation in the sports context and choking-like performances [20,21]. Yips-affected golfers tend to be anxious in the putting situation about their performance because of having experienced the putting yips many times [12]. They might expect the occurrence of the twists and jerks in the wrist and therefore attempt to consciously control the movement to prevent the yips. This movement strategy has been described as the paradox of control [22] or the vicious circle of yips-affected golfers [7], and might lead to the opposite, the occurrence of the yips.

Individual differences in athletes in terms of the dispositional tendency to reinvest also influence the likelihood of reinvestment incidences [23]. The tendency to reinvest can be measured with the Movement-Specific Reinvestment Scale consisting of two subscales [14]. The subscale *movement self-consciousness* deals with concern about one’s own movement style. The subscale *conscious motor processing* measures the cognitive reflection of the movement process. The Movement-Specific Reinvestment Scale indicates the athlete’s tendency to reinvest. It does not indicate whether an athlete actually reinvested or not in a specific situation. In a symposium contribution, it has been claimed that yips-affected athletes have a higher tendency to reinvest than unaffected athletes [24]. Klämpfl et al. [6], however, found no difference in reinvestment between affected and unaffected golfers, probably due to different yips criteria.

The validity of reinvestment as the underlying mechanism can be tested by manipulating the focus of attention, by either focusing on a skill to provoke reinvestment or distracting attention from performing a skill to prevent reinvestment. Focus of attention was previously manipulated by instructions (e.g., 25) and by the application of dual-task paradigms (e.g., 26). The application of dual-task paradigms has the advantage of providing an indication about the effectiveness of the attentional manipulation [18]. Dual-task paradigms involve a primary task—here, golf putting—and a secondary task that is created to direct the participants’ attention—in the following experiment, a tone-monitoring task (e.g., 17). The performance in the secondary task indicates if the participants applied the desired attention. A recent study showed by applying a dual task paradigm that yips-unaffected expert golfers performed worse in a skill-focus condition than in the extraneous condition [26].

### Aim of the Study

The present study aimed to confirm that reinvestment also leads to the occurrence of the yips, which would have important implications for designing effective interventions. This is the first study that used a dual-task paradigm in a putting experiment in yips-affected golfers. We manipulated the conditions to either direct their attention to their own motor action (skill-focus condition) or distract them from the own skill execution (extraneous condition). We hypothesized that yips-affected golfers would exhibit reduced yips behavior, assessed by performance and movement variability over repeated putts, in the extraneous condition compared to the skill-focus condition, if reinvestment is responsible for the occurrence of the yips. We also explored if the tendency to reinvest can explain yips behavior in the two attention-manipulating conditions.

## Methods

### Ethics Statement

Ethical clearance to conduct the study was provided by the ethics committee of the German Psychological Society (August 14^th^ 2010) and the ethics board of the German Sport University Cologne (October 27^th^ 2009). They approved the following consent procedure: Before the participants gave their written consent, they were informed about the purpose of the project, the procedure of the study, the privacy regulation, the opportunity to quit the participation any time without receiving any consequences, and that their data will be deleted when requested.

### Participants

**Figure 1:**
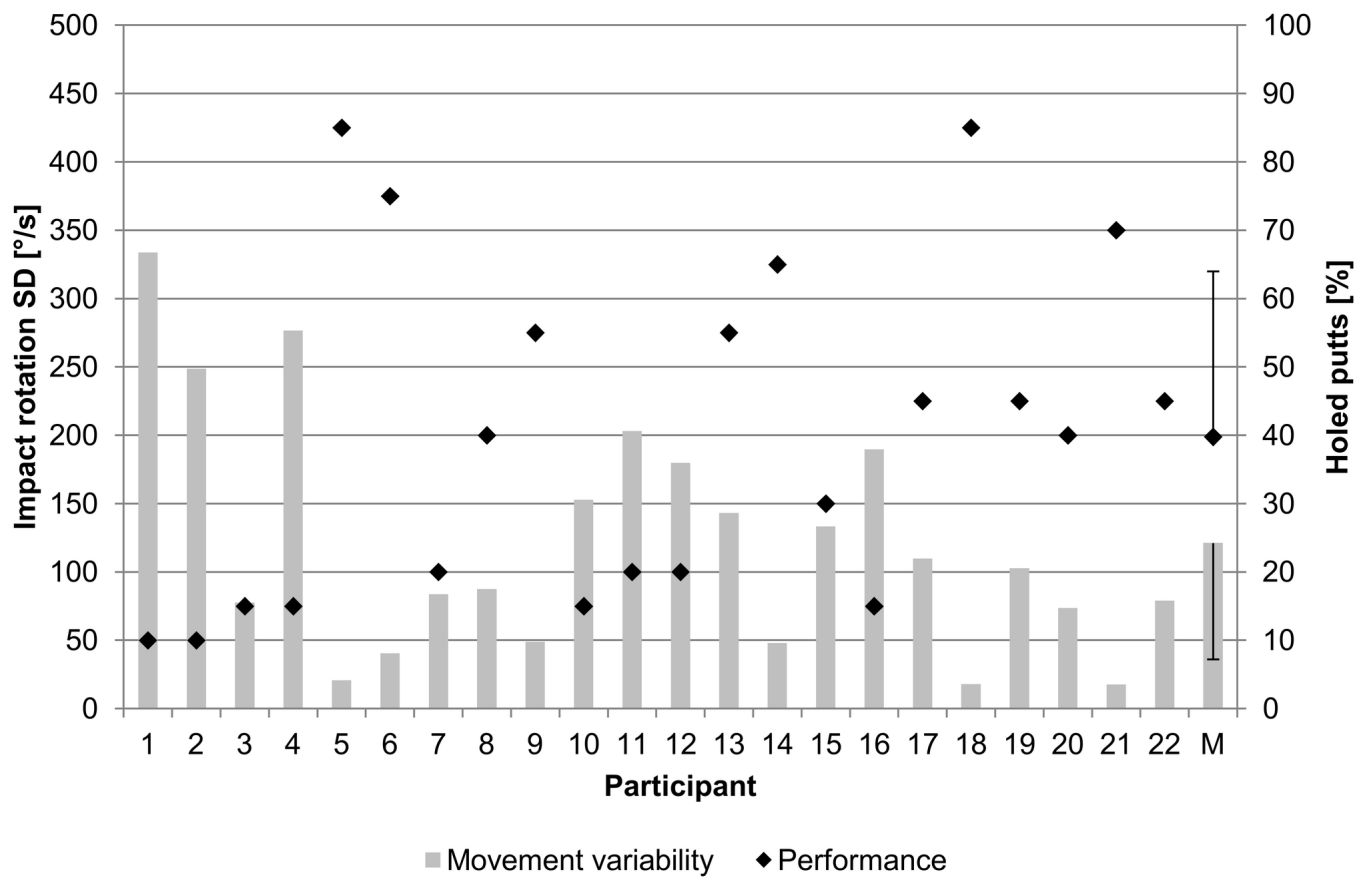
Movement variability and performance of the sample in the pretest. The excluded participants (5, 18, 21) by video rating also exhibited a low movement variability. The standard deviation for the mean (M) is indicated as error bars.

Twenty-two golfers suspected of being yips affected were recruited from previous studies and from screening of more than 200 golfers in regional golf clubs. They were recruited when they fulfilled the yips criterion, consisting of observed involuntary movements during the execution of one-handed putts, specifically before ball contact [6]. Shortly before the actual experiment, they had to perform 20 putts with their dominant arm in a pretest, while the frontal plane of the golfers was videotaped (mild and severely yips-affected putts: Video S1 and Video S2). After the experiment, the videos were rated as either yips-affected or unaffected. The inter-rater reliability of two independent raters who were trained in observing the yips was 82%. The raters watched the differently rated videos again until consensus was reached. Three golfers had to be subsequently excluded. The movement variability of the golfers in the pretest supports the video ratings (Figure 1).

Nineteen yips-affected golfers (15 males, 4 females; age: *M* = 55.9 years, *SD* = 13.0; handicap: *M* = 21.9, *SD* = 12.0; golfing experience: *M* = 11.2 years, *SD* = 13.1; golfing frequency: *M* = 8.0 hr/week, *SD* = 4.8) were included in the study. Two golfers did not have a handicap due to being either a professional golf teacher or an unregistered golfer. The dominant arm of all participants in putting was the right arm. We estimated their experience in golfing only with the dominant arm as the level of automatization of the skill has an influence on the detrimental effect of reinvestment. The one-handed putting experience was low (*M* = 0.7, *SD* = 0.9), estimated on a 5-point Likert scale from 0 (*no experience*) to 4 (*I only putt with one arm*).

### Apparatus

The participants putted indoors at a golf club on an artificial putting green consisting of connected square turf tiles measuring 0.25 m^2^ each. The hole was placed at a distance of 1 m, representing an easy putt for unaffected golfers but a difficult putt for affected golfers. The participants putted with standardized blade putter (Odyssey, Callaway Golf Europe Ltd., Surrey, U.K.) and balls (Srixon AD333, Srixon Sports Europe Ltd., Alton, U.K.). Kinematics of the putter were obtained with the ultrasound-based SAM PuttLab Pro Wireless 2010 System (Science&Motion GmbH, Munich, Germany) at a rate of 70 Hz. Data were processed and analyzed using the SAM PuttWare Pro (Version 2010–034) software. A video camera captured the frontal plane of the arms and the lower body of the golfers, including the putter and the ball. Tones were presented with Inquisit 3 (Millisecond Software, Seattle, Wash.) via loudspeakers. Statistical analyses were performed with SPSS 20 (IBM Corporation, Armonk, N.Y.). For the a priori sample size estimation and the calculation of the post hoc statistical power, we used G*Power 3 [27].

### Pretest and Putting Conditions

Participants had to putt with only their right arm in a pretest and two attention-manipulating conditions, namely, a skill-focus and an extraneous condition. Twenty putts of each participant were recorded for the pretest and for each of these conditions. In all conditions, one of four tones (250 Hz, 300 Hz, 500 Hz, and 550 Hz) was randomly presented at a random point in time for a duration of 100 ms during the execution of the putt, similar to in Castaneda and Gray [17]. We included two additional tones (300 Hz and 550 Hz) in an attempt to balance the difficulty of the secondary-task conditions (described below). If successfully managed, task difficulty cannot be the reason for performance differences in the secondary tasks. The pretest represented a yips test, ensuring that only yips-affected golfers participated in the study. In the pretest, the participants were instructed to just ignore the tones. The skill-focus condition and the extraneous condition included additional tasks as in previous studies (e.g., 17), which showed their effectiveness in manipulating attention in the desired direction.

#### Skill-focus condition

**Figure 2:**
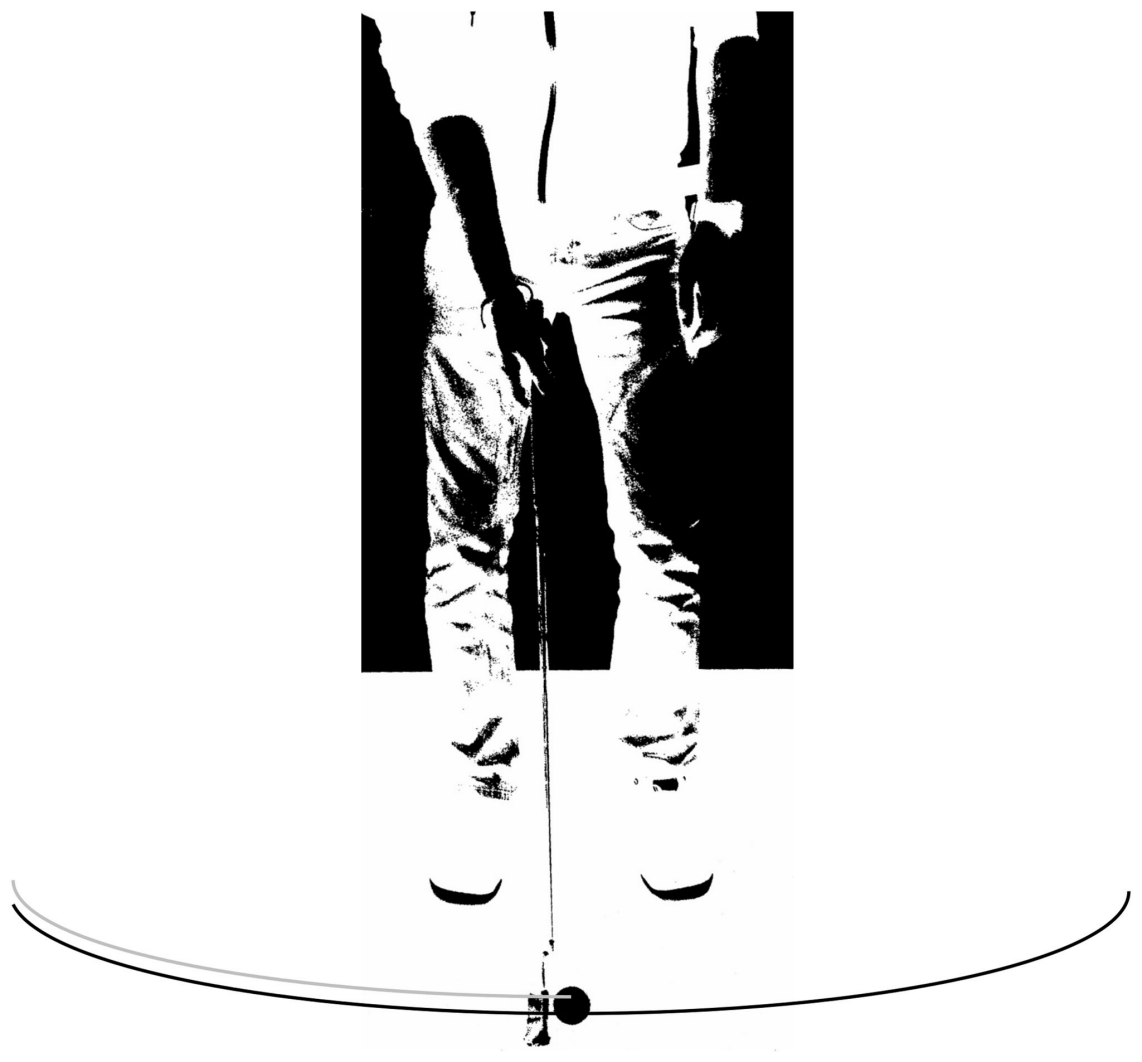
Answer sheet for the secondary task in the skill-focus condition. After the execution of the putt, the participants had to indicate as accurately as possible where the lower arm was located on the swing curves during the execution, when the tone was presented. The gray curve indicates the backswing. The black curve indicates the forward swing.

Participants were told to perform an additional task while they were putting to ensure that they directed their attention to the skill. They were instructed to focus on their right lower arm, the putting arm. After the execution of the putt, they had to indicate as accurately as possible on a sheet of paper where the lower arm was located during the execution, when the tone was presented. The sheet showed a standardized image of the putting swing from the same perspective as the camera and included separate lines for the backswing and forward swing (Figure 2).

#### Extraneous condition

Participants had to perform an additional task while they were putting to direct their attention away from their own skill. They were instructed to focus on the tones. After the execution of the putt, they had to indicate as accurately as possible whether the tone was low or high. Before putting, participants listened to two low tones (250 Hz, 300 Hz) and two high tones (500 Hz, 550 Hz) two times. Four tones were chosen to make the secondary task in the extraneous condition more difficult than if only two tones were presented, as in Castaneda and Gray [17], in an effort to balance the difficulty of the secondary tasks in each condition.

### Measures

Movement variability and putting performance were measured to evaluate the change in yips behavior, as proposed by Klämpfl et al. [6]. Movement variability characterizes the uncontrollability of the movement and was expressed as the standard deviation of the rotation of the putter at ball impact over the 20 trials in each condition. Putting performance was indicated as the percentage of putts holed in each condition.

Secondary-task performance was measured as the percentage of correct answers. In the skill-focus condition, the accuracy rate referred to the location of the lower arm during the three swing phases of the putt (backswing, forward swing to ball impact, and forward swing after ball impact), when the tone was presented. In the extraneous condition, the accuracy rate of judging the tone as either high or low was derived.

Furthermore, the tendency to reinvest was measured via the Movement-Specific Reinvestment Scale (German translated version as in Klämpfl et al. [6]; original version from Masters and Maxwell [14]) consisting of nine items in total. Sample items include “I am always trying to think about my movements when I carry them out” for the *conscious motor processing* subscale and “I am self-conscious about the way I look when I am moving” for the *movement self-consciousness* subscale. Participants had to indicate their agreement on a Likert scale from 1 (*strongly disagree*) to 6 (*strongly agree*). The internal reliability of the German validated scale was acceptable (Cronbach’s alpha = .73 and .67).

### Procedure

Before putting, the participants gave their informed consent and filled in the Movement-Specific Reinvestment Scale. All participants started with the pretest. They subsequently putted in the skill-focus and extraneous conditions as described above in a counterbalanced order. Participants performed two practice putts prior to the actual measurements to familiarize themselves with the task in each condition. Feedback about the accuracy of the participants’ answers on the secondary task was given only for the practice putts.

### Statistical Analysis

A repeated-measures multivariate analysis of variance (MANOVA) with condition (skill-focus, extraneous) as a within-group factor was used to evaluate differences between the skill-focus and extraneous putting conditions. The significance level was set to .05. We report the Pillai–Spur *F* statistics. We assumed that the manipulation would have the same large effect on yips-affected golfers as on unaffected golfers, as in Beilock and Gray [26] (d = .92). Therefore, an effect of .65 (Cohen’s *f*) and a power of .96 would require a sample size of 18 participants. One outlier was identified but was kept because there were no changes in the results when removed for the parameters included in the MANOVA and there were no missing values. An initial exploration of the data revealed an unexpected carry-over effect between the putting conditions. Split-half reliabilities (odd vs. even, first half vs. second half) of the performance and the kinematic parameter were calculated for the pretest and putting conditions via the formula devised by Kristof [28], which is preferred for small samples. Separate linear regression analyses were conducted with the main reinvestment scale and the subscales to test if these variables could predict the yips behavior in the putting conditions. To do this, the carry-over effect between the putting conditions was removed, and subsequently, the differences in performance and movement variability between the skill-focus and the extraneous condition were used as the dependent variables. Correlations of all variables (Table S1) used and the SPSS data set (Data Set S1) can be found in Supporting Information.

## Results

### Reliability and Manipulation Check

Before testing the main hypotheses, we ensured that the measures we used were reliable and that the manipulation was successful. There was sufficient reliability of the derived measures in the pretest and in the attention-manipulating conditions (Table 1). The reliability values were relatively stable between the conditions and between the split-half methods, specifically odd versus even and first half versus second half. Moreover, we made sure that the manipulation directing the focus of attention was successful. In this context, the accuracy in the secondary task in the skill-focus condition, in which the participants had to indicate the location of the lower arm during the putting swing, when the tone was presented, was on average 77.6% (SE = 3.1) and ranged between 45 and 95%. The participants’ accuracy in the extraneous condition, where they had to determine the frequency of the tone (low or high), was on average 96.8% (SE = 1.2) and ranged between 80 and 100%.

**Table 1:** Reliability of measures used in the pretest and putting conditions.

Measure	Split-half method	Reliability		
		Pretest	Skill-focus condition	Extraneous condition
Putting performance	First half vs. second half	.79	.92	.89
	Odd vs. even	.82	.95	.92
Impact rotation SD	First half vs. second half	.91	.94	.95
	Odd vs. even	.94	.92	.99

### Putting Conditions

**Figure 3:**
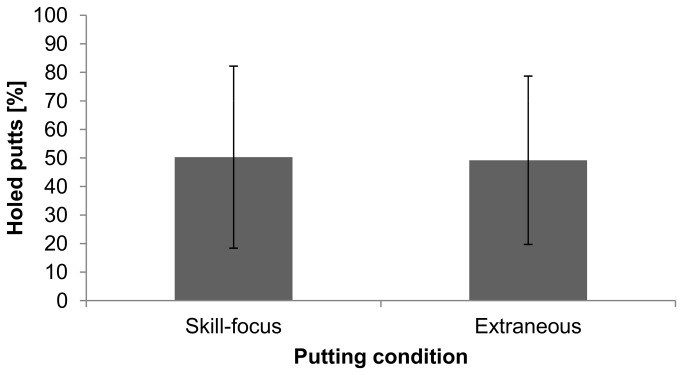
Performance in the skill-focus (*SE* = 7.3) and extraneous (*SE* = 6.8) conditions. Error bars indicate the standard deviations of the mean.

**Figure 4:**
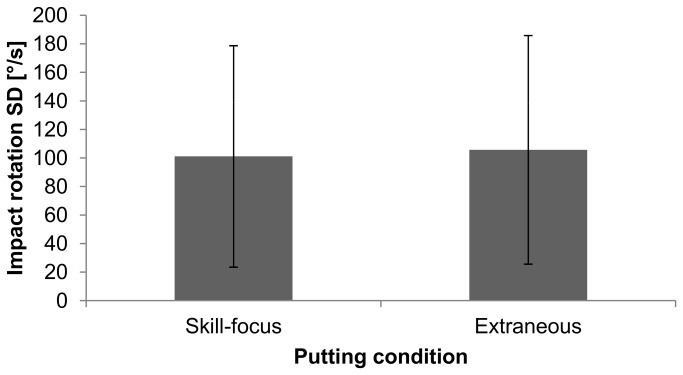
Movement variability in the skill-focus (*SE* = 17.8) and extraneous (*SE* = 18.4) conditions. Error bars indicate the standard deviations of the mean.

The mean and standard deviations of movement variability and performance are indicated in Figures 3 and 4. The movement variability values ranged between 14.8 and 251°/s in the skill-focus condition, and 7.9 and 280°/s in the extraneous condition. Performance ranged between 5 and 100% in the skill-focus condition and between 0 and 95% in the extraneous condition. The one-factorial (condition: skill-focus, extraneous) MANOVA with putting performance and movement variability as dependent variables showed no effect of condition, *F*(2, 17) = 0.05, *p* > .05, η^2^ = .006, meaning that the performance and movement variability of the yips-affected golfers did not differ between the skill-focus condition and the extraneous condition. The post hoc estimated power of the test (with Cohen’s f = 0.078) was 7.4%.

### Reinvestment Tendency

The participants scored on average 3.2 (SD = 1.0) on the main reinvestment scale, 3.0 (SD = 1.1) on the movement self-consciousness subscale, and 3.4 (SD = 1.1) on the conscious motor processing subscale. Neither the main reinvestment scale score nor the subscale scores could predict yips behavior, more specifically, performance and movement variability, in the putting conditions (*R* ^2^ < .01, *F* < .16, *p* > .05). In addition, there were no significant correlations between the reinvestment scales and yips behavior measures, such as movement variability and performance in the putting conditions (Table S1).

## Discussion

This is the first study aimed to empirically confirm with a laboratory experiment that there is a link between the yips and reinvestment. Although previous studies claimed an existing link, the data in the present study could not confirm this. It was hypothesized that yips-affected golfers would exhibit reduced yips behavior—specifically higher performance and lower kinematic inconsistency—in the extraneous condition than in the skill-focus condition if reinvestment is responsible for the occurrence of the yips. However, there was no difference in yips behavior between the two dual-task-based conditions created to direct the focus of attention. Referring to previous studies (e.g., 17), the performances in the secondary tasks indicate that the desired manipulation of the focus of attention was successful. In addition, the measures used exhibited sufficient reliability. The present study did not achieve sufficient test power with yips-affected golfers, although previous studies did with a similar sample size consisting of unaffected golfers.

In previous studies [16,26], yips-unaffected expert golfers experienced performance decrements in a putting task when their attention was directed to the skill. The same was not observed in the present study for yips-affected golfers. We are confident that the yips-affected golfers in the sample had sufficient experience in putting to potentially suffer from reinvestment. The participants, however, had little experience with the experimental task consisting of one-handed putts with the dominant arm from a distance of one meter, which represents an easy task for unaffected golfers. If the participants could therefore be seen as novices in this task, then they could have potentially benefitted more from focusing on a skill, as previously shown for yips-unaffected less skilled athletes [16–18], but this was not the case.

An indication that reinvestment plays a role in the occurrence of the yips was also not obtained by measuring the tendency to reinvest. Contradictory findings existed about the tendency to reinvest in yips-affected athletes. Rotheram et al. [24] found higher scores for yips-affected athletes, whereas Klämpfl et al. [6] did not find any difference between yips-affected and unaffected golfers. The score on the main reinvestment scale (*M* = 3.2) in the present study indicates a medium reinvestment level and is similar to that reported in Klämpfl et al. [6] because the same yips criterion and part of the same sample were used. The regression analysis revealed that the tendency to reinvest could not explain the yips behavior in the two attention-manipulating conditions. According to reinvestment theory, higher reinvesters should have benefitted more from the extraneous condition, but this was not supported by the data.

According to the results, reinvestment or the attempt to consciously control their own movements did not appear to be responsible for the occurrence of the yips. Nevertheless, this is the first study to empirically test the relevance of reinvestment for the yips. On the one hand, more studies with different designs are needed to confirm the results. For instance, intervention studies implementing methods reported to prevent reinvestment, such as implicit motor learning [15], distraction training (e.g., 29), or the learning of preperformance routines (e.g., 30), might reveal if long-term treatment has a positive effect on the yips. On the other hand, other possible yips etiologies, such as the yips as a neurological disorder (e.g., 3) or as a conditioned reaction [7], need to be further tested. The latter, yips as a conditioned reaction, seems to be especially promising, as the yips was not influenced by the attention manipulation in the present study and there was no indication of a neurological origin of the yips in Klämpfl et al. [6]. Future studies might search for a conditioned stimulus of the yips with contextual manipulations, or for the role of vision and anticipation of the putter–ball impact, or they might even find a way to teach the yips. In addition, the application of brain imaging measurement systems (e.g., magnetic resonance imaging), as have been used for unaffected golfers [31], could reveal more insights into the etiology of the yips.

Future investigations might consider the following limitations of the present study. A baseline condition consisting of a single task without an attentional manipulation is missing. Such a task would indirectly indicate what kind of attentional focus the yips-affected golfers are used to applying, when performance is compared to that in the attentional manipulated conditions (e.g., 26) or when participants are asked post hoc about their applied focus of attention [32]. Finally, no qualitative data was derived to get information about possible events (e.g., significant choking experience) that resulted in reinvestment and possibly onset of the yips.

## Conclusions

 The present study was the first to empirically test the link between reinvestment and the yips with a laboratory experiment. No support for the speculations of previous studies [6,13,24] was found, as no link between reinvestment and the yips was shown. Furthermore, the tendency to reinvest did not explain yips behavior. Therefore, reinvestment might be not responsible for the occurrence of the yips. Future investigations could conceptually replicate these findings by using different designs, such as intervention studies. They could test the validity of alternative explanations for the occurrence of the yips to provide a better understanding of the yips and to appropriately treat affected athletes.

## Supporting Information

Video S1 **Mild yips.**
(MP4)

Video S2 **Strong yips.**
(MP4)

Table S1 **Correlation matrix for all variables used in the study.**
(PDF)

Data Set S1 **Data set.**
(SAV)
