# Supplementary material for: Reinvestment – the Cause of the Yips?
Source: PLoS One. 2013 Dec 5;8(12):e82470. doi: 10.1371/journal.pone.0082470 (PMC3855447; doi:10.1371/journal.pone.0082470)
Supplement: Table S1 — Correlation matrix for all variables used in the study. (PDF) [file pone.0082470.s003.pdf]

Table S1. Correlation matrix for all variables used in the study.

Measure	Age	Hcp	Golf experience	Golf frequency	MSRS	Conscious motor processing	Movement self-consciousness	Performance, skill focus	Performance, extraneous	Performance, skill-focus task	Performance, extraneous task	Impact rotation *SD,* skill focus	Impact rotation *SD,* extraneous
Age	–												
Hcp	-.52*	–											
Golf experience	.34	-.46	–										
Golf frequency	.68**	-.47	.17	–									
MSRS	-.25	.11	-.11	-.05	–								
Conscious motor processing	-.24	.06	-.10	-.10	.89**	–							
Movement self-consciousness	.21	.12	-.11	-.01	.92**	.63**	–						
Performance, skill focus	-.10	-.12	.41	-.27	.10	.11	.10	–					
Performance, extraneous	-.10	-.03	.44	-.21	-.01	.06	-.01	.72**	–				
Performance, skill-focus task	-.19	-.14	.01	-.07	.22	.45	-.01	.04	.10	–			
Performance, extraneous task	.03	-.27	.21	.27	.09	.06	.10	.17	.41	.07	–		
Impact rotation *SD,* skill focus	.38	-.08	-.24	.44	-.26	-.20	-.26	-.89**	-.66**	-.13	-.10	–	
Impact rotation *SD,* extraneous	.27	-.11	-.29	.36	.01	.09	.01	-.67**	-.91**	.07	-.36	.70**	–

Note. Hcp: Handicap. MSRS: Movement-Specific Reinvestment Scale..
*significant at the level of .05; **significant at the level of .01
